# Evaluation the oral hygiene conditions, oral Candida colonization and salivary *Streptococcus mutans* and *Lactobacilli* density in a group of β-thalassemic children and adolescence

**DOI:** 10.4317/medoral.23024

**Published:** 2019-10-27

**Authors:** Hüseyin Karayilmaz, Hande Yalçin-Erman, Özge Erken-Güngör, Zeynep Öztürk, Rasih Felek, Alphan Küpesiz

**Affiliations:** 1 Akdeniz University, Faculty of Dentistry, Department of Pedodontics; 2 Antalya Oral and Dental Health Center; 3 Akdeniz University, Faculty of Medicine, Department of Pediatric Hematology-Oncology; 4 Akdeniz University, Faculty of Dentistry, Department of Microbiology

## Abstract

**Background:**

In this study, the prevalence and distribution of dental caries and oral hygiene conditions in a group of patients with β-TM are evaluated and the results compared to age-and gender-matched healthy patients. In addition, oral candida colonization and the density of *Streptococcus mutans* (*S.mutans*) and *Lactobacilli* in the total saliva are assessed.

**Material and Methods:**

This study involved 59 β-TM patients between 6-16 years old (mean:11.59±3.22), who applied to the Department of Pedodontics, Faculty of Dentistry, Akdeniz University, with ongoing follow-up, treatment and regular blood transfusions. All enrolled patients were diagnosed with β-TM by the Department of Pediatric Hematology and Oncology, Faculty of Medicine, Akdeniz University. As a control group, age-and gender-matched healthy 50 patients were included to the study.

**Results:**

Plaque (*p*=0.001), DMFT (*p*=0.009) and DMFS (*p*=0.039) indices were significantly higher in the β-TM patients, whereas, the oral hygiene status was significantly lower (*p*=0.004). Saliva buffering capacity average was insignificantly but slightly more in β-TM patients(*p*=0.131). While *S.mutans* values were significantly higher in the β-TM patients (*p*=0.002), no significant difference was found in the *Lactobacillus* (*p*=0.131) and *Candida* values (*p*=0.33).

**Conclusions:**

DMFT, DMFS, Plaque and oral hygiene indices and *S.mutans* values were found significantly different in β-TM patients than healthy, control group patients, in this study.

** Key words:**Thalassemia major, DMFT, S.mutans, Lactobacilli, Candida.

## Introduction

Hemoglobin is the oxygen-transport protein in the red blood cells. Hemoglobin A is the major hemoglobin, comprising about 97% of the total hemoglobin. The structure of hemoglobin A typically consists of two alpha (α) and two beta (β) globin chains (1,2). The thalassemias are a group of genetic disorders characterized by decreased or absent production of globin chains, leading to microcytic anemia. The thalassemias are categorized according to the defective globin chain: -thalassemia and -thalassemia. -thalassemia is the most common genetic disorder worldwide (1-3), and is widespread in the Mediterranean countries, the Middle East, Central Asia, India, South China, the Far East, and on the northern coasts of Africa and South America. -thalassemia is divided into three groups; thalassemia minor, thalassemia intermedia and thalassemia major (TM). Thalassemia minor is the carrier form of the disease, and carriers have asymptomatic mild-to-moderate microcytic anemia. Thalassemia intermedia patients present less severe clinical features than TM patients (1-3), and do not need regular blood transfusions, as they usually have hemoglobin levels of 7–10 g/dl (4). β-TM is also known as Cooley anemia or Mediterranean anemia. Patients with β-TM have severe microcytic hypochromic anemia, due to the reduced or absent production of β-globin chains, requiring repeated erythrocyte transfusions (2-4).

Children born with β-TM only show symptoms during the first year of life when adult hemoglobin has replaced fetal hemoglobin (5-8). Peripheral anemia arising from the disease sends signals to the bone marrow to increase the production of red blood cells, but the production of red blood cells is abnormal. With time, the marrow cavities expand (e.g., skull bones, facial bones, ribs), leading to characteristic facial features (referred to as “chipmunk” facies or “rodent-faced”) and radiographic findings. If the disease is not treated, death can occur from anemia and congestive heart failure. The proper treatment for these patients includes routine blood transfusions and other therapies to survive (5-8).

The most common oro-facial features among thalassemia patients, because of intense compensatory hyperplasia of the marrow and expansion of the marrow cavity, are skeletal malocclusion, maxillary protrusion, mandibular atrophy, increased overjet, anterior open bite, prominence of the malar eminence, flat nose, lateral displacement of orbits and frontal bossing (5-8). Other dental features include flaring and spacing between maxillary incisors, cuspidal short roots, taurodontism, attenuated lamina dura and thin mandibular cortex (5-8). Additionally, thalassemic patients may have other oral symptoms, such as dental caries, due to the neglect of oral hygiene but also because of the decreasing concentrations of phosphorous and immunoglobulin A (Ig A) in saliva (5,6,8-15).

TM is one of the most common genetic disorders causing public health concern worldwide, including the Mediterranean region of Turkey. Nonetheless, a review of the literature demonstrates a lack of comprehensive studies regarding oro-facial features, dental caries and oral hygiene conditions of patients with TM.

In this study, the prevalence and distribution of dental caries and oral hygiene conditions in a group of patients with β-TM are evaluated and the results compared to age- and gender-matched healthy patients. In addition, oral candida colonization and the density of *Streptococcus mutans* (*S. mutans*) and *Lactobacilli* in the total saliva are assessed.

## Materials and Methods

This study was supported by Akdeniz University, The Scientific Research Projects Coordination Unit, under the Project No: 2014.01.0151.002.

This case-control study involved 59 β-TM patients between 6 and 16 years old, who applied to the Department of Pedodontics, Faculty of Dentistry, Akdeniz University, with ongoing follow-up, treatment and regular blood transfusions. All enrolled patients were diagnosed with β-TM by the Department of Pediatric Hematology/Oncology, Faculty of Medicine, Akdeniz University and had no systemic diseases, except TM. Patients who used antibiotics or anti-fungal medicines within the last 3 months and having splenectomy history, were excluded. As a control group, 50 patients between 6 and 16 years old, without any systemic disease, who applied to our clinics for routine dental treatments and did not use antibiotics and/or anti-fungal medicines within the last 3 months, were included. None of the patients enrolled in the study and control groups were smokers. Patients and parents were informed, and their written approvals were taken. Evaluations were carried out in the morning before any dental treatment was applied.

The Akdeniz University Research Ethics Committee approved the study (decision dated 12.02.2014 and numbered 113).

- Evaluation of Oral Hygiene 

Oral hygiene (debris, calculus) status of the patients in the study and control groups was evaluated by the Simplified Oral Hygiene Index (16), which has two components: The Debris Index and the Calculus Index. Each of these indices is based on numerical determinations, representing the amount of debris or calculus found on the preselected tooth surfaces (16).

Plaque test (CRT Plaque Test, Ivoclar Vivadent, Liechtenstein) solution (a fluorescent disclosing liquid for the exposure of plaque) was used to evaluate the patients’ plaque index, according to Silness and Löe (17).

A standard CRT buffer kit (CRT Buffer, Ivoclar Vivadent, Liechtenstein) was used to determine the saliva buffering capacity of the patient, by means of a test strip featuring a specific indicator system [yellow/brown: pH<4.0 (low), green: pH<4.5-5.5 (medium), blue: pH>6.0 (high)].

The tooth-level indices (DMFT/dft) and surface-level indices (DMFS/dfs) were calculated. The DMFT and DMFS indices are applied to the permanent dentition and are expressed as the total number of teeth or surfaces that are decayed (D), missing (M) or filled (F), in an individual, respectively. When written in lowercase letters, the dft and dfs indices are a variation that is applied to the primary dentition. Missing primary teeth should be ignored, because of the difficulty in distinguishing between teeth extracted due to caries and those that have naturally exfoliated.

- Microbiological Evaluation 

In the morning, saliva samples stimulated with paraffin gum were collected from patients in the study and control groups, for microbiological evaluation. The patients were not allowed to eat or drink anything, chew any chewing gums, smoke, brush their teeth and use any mouthwashes, at least 1 hour before the test was conducted. Saliva samples, placed in sterile boxes, were homogeneously spread onto test kits (CRT Bacteria Refill, Ivoclar Vivadent, Liechtenstein) that have a selective medium for *S. mutans* and *Lactobacillus* on the respective sides. After incubation at 37°C for 48 hours, *S. mutans* and *Lactobacillus* were enumerated, according to the manufacturer’s instructions.

- Evaluation of *Candida*l Colonization 

Swab samples, which were taken with an applicator from the throat and cheek mucosa of study and control groups, were cultivated on a specific media with a loop (BD Sabouraud agar, including gentamicin and chloramphenicol), and incubated at 37°C for 24–28 hours. Samples were taken from single colonies in the medium with a loop, and examined under a light microscope, by diluting with serum. Samples that were diagnosed with *Candida* were subcultured onto corn meal agar, Sabouraud dextrose agar and CHROMagar media. *Candida* spices were defined by API 20C AUX kits (BioMérieux, Marcy l'Etoile, France).

- Statistical Evaluation

Quantitative variables among the groups were compared using the Student’s t-test, for parametric data. Nonparametric data were analyzed by the Kruskal–Wallis and Mann-Whitney U tests. Qualitative variables were examined with the Kruskal–Wallis, Mann-Whitney U, chi-square and exact Fisher tests, respectively. Statistical significance was assumed at *p*<0.05. All calculations were performed using SPSS 15.0 (SPSS Inc., Chicago, IL, USA).

## Results

The study group consisted of 59 β-TM patients (36 girls, 23 boys) aged between 6 and 16-years (mean: 11.59±3.22 years). The control group consisted of 50 patients, whose ages were the same range as those in the study group (mean: 11.06±2.86 years). The study and control groups were divided into subgroups, according to permanent and mixed dentition periods, for evaluation.

**Figure 1:**
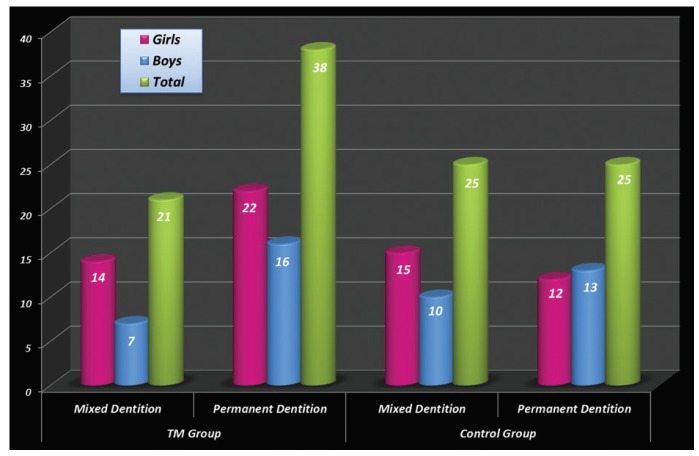
Distribution of β-TM and control group patients according to gender and dentition

In β-TM patients, the 21 patients in the mixed dentition subgroup were aged between 6 and 11 years (average: 8.04±1.71 years) while the 38 patients in the permanent dentition subgroup were aged between 10 and 16 years (mean:13.55±1.91 years) (Fig. 1). In the control group, the 25 patients in the mixed dentition subgroup were aged 6–9 years (average: 7.4±1.04 years) while the 25 patients in the permanent dentition subgroup were 12–16 years old (mean: 12.72±0.93 years) (1). 

The DMFT, DMFS, dft and dfs ratios obtained from the β-TM and control group patients (and from subgroups, mixed and permanent dentition) have been summarized in Table 1, and the oral hygiene statuses, plaque indices and saliva buffering capacities have been summarized in Table 2. In comparison to the control group, the plaque, DMFT and DMFS indices were significantly higher in the β-TM patients (plaque index: *p*=0.001; DMFT index: *p*=0.009; DMFS *p*=0.039) (Table 1), whereas, the oral hygiene status was significantly lower in β-TM (oral hygiene index: *p*=0.004). Although the saliva buffering capacity average was slightly more in β-TM patients than the control group, the difference was not significant (saliva buffering capacity: *p*=0.131) (Table 2).

When gender differences in the β-TM patients and control group were considered, only the plaque indices of male β-TM patients were found to be higher (*p*=0.002). No significant relationship was found in other parameters (*p*>0.05) (Table 2).

**Table 1:**
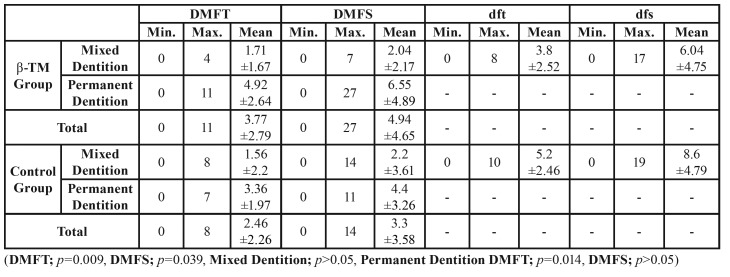
The results of DMFT, DMFS, dft, and dfs indexes.

**Table 2:**
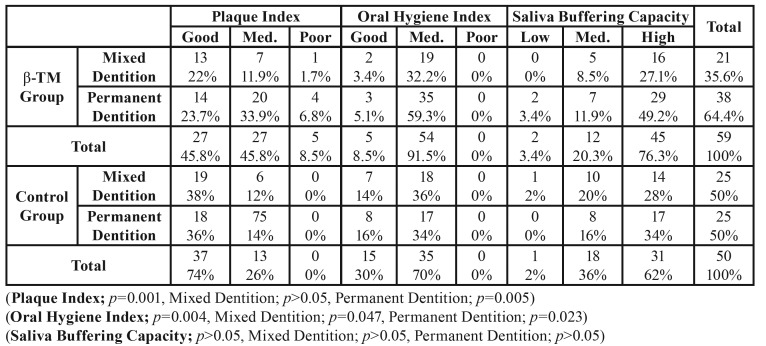
The results of Plaque Index, Oral Hygiene Index and Saliva Buffering Capacity.

- Mixed Dentition

A significant difference was only observed in the oral hygiene index, among the parameters examined during the mixed dentition period. The oral hygiene index was lower in β-TM patients than control group patients (oral hygiene index: *p*=0.047) (Table 2).

- Permanent Dentition 

Plaque index, oral hygiene index and DMFT index values were shown to be significantly higher in β-TM patients than the control group, during the permanent dentition period (DMFT index: *p*=0.014; plaque index: *p*=0.005; oral hygiene index: *p*=0.023). There was no significant difference in the DMFS index and saliva buffering capacity between the β-TM and control patients (DMFS: *p*=0.058; saliva buffering capacity: *p*=0.567) (Table 1, Table 2).

- Microbiological Evaluation 

*S. mutans* and *Lactobacillus* values obtained from β-TM and control group patients (and from subgroups, mixed and permanent dentition) have been summarized in Table 3. While *S. mutans* values were significantly higher in the β-TM patients than the control group (*S. mutans*: *p*=0.002), no significant difference was found in the *Lactobacillus* values between these two groups (*Lactobacillus*: *p*=0.131) (Table 3). A statistically significant effect of gender was not evident (*p*>0.05).

- Mixed Dentition

No significant relationship was seen in the *S. mutans* and *Lactobacillus* values between the two groups during the mixed dentition period (*S. mutans*: *p*=0.86; *Lactobacillus*: *p*=0.418) (Table 3).

- Permanent Dentition

*S. mutans* values were statistically higher in β-TM patients in the permanent dentition period when compared to the control group (*p*=0.008). No significant difference in *Lactobacillus* values appeared between the two groups (*Lactobacillus*: *p*= 0.507) (Table 3).

**Table 3:**
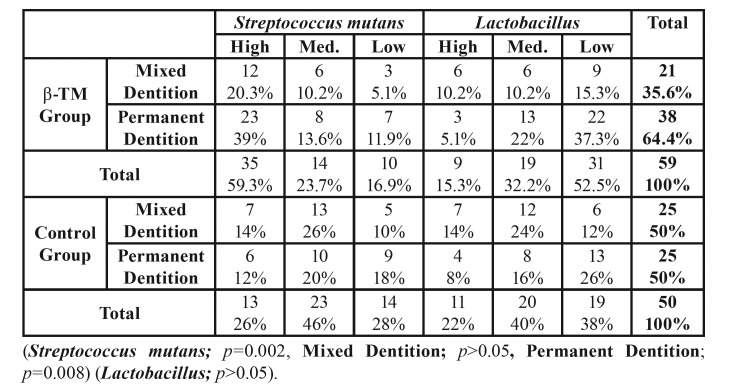
Distribution of *Streptococcus mutans*, *Lactobacillus values*.

- Evaluation of *Candida*l Colonization 

Although, it was not observed in 44 of the β-TM patients (74.6%), *Candida* was seen only in 15 patients. After the typing process, **Candida* albicans* was detected in 14 patients (23.7%), and *Candida* glabrata was identified in 1 patient (1.7%). In the control group, *Candida* occurred in 33 out of the 50 patients (66%). **Candida* albicans* was found in 16 of these patients and *Candida* parapsilosis in 1 patient, following the typing process.

There was no significant correlation in the *Candida* values between the study and control groups (*p*=0.33). Given the low *Candida* evaluation rate of patients in the study and control groups, the patients in the subgroups (mixed and permanent dentition) were not evaluated.

## Discussion

Oral and dental problems and their treatments affect the general health of the human body. Especially in people with systemic problems, oral and dental issues must be evaluated, and certain procedures must be carefully followed for treatment. The oral hygiene status must be examined, and decay activities of the patients must be defined in at-risk patients, such as TM patients.

A limited number of studies are currently available on the oral and dental problems of TM patients. Whereas some of the studies asserted that the decay frequency of TM patients was higher when compared to healthy people, some reports claim that there were not any differences (6,9-11).

Lugliè *et al*. (9) evaluated the oral hygiene indices, *S. mutans* levels and DMTF indices of 18 TM patients aged between 23 and 31 years, and 18 control group patients in the same age range, in 2002. The authors stated that *S. mutans* values were significantly higher in TM patients when compared to control group, and, although the DMFT indices were higher in TM patients (TM: DMFT=10.3; control: DMFT=9.4), this difference was not significant. In addition, no statistically meaningful difference in the oral hygiene indices between the two groups was determined while plaque existence was higher in the control group patients.

In the same year, Al-Wahadni *et al*. (10) analyzed DMFT, plaque and gingival index values in a study of 61 β-TM patients and 63 control patients aged 6-12 and 13-18-years. The results revealed that the DMTF indices were significantly higher in β-TM patients for both of the age groups. Plaque and gingival index values were also higher in β-TM patients, in both age groups, but it was not significant.

In 2001, Hattab *et al*. (11) examined the plaque scores and DMFT/dmft values of 54 TM patients aged between 6 and 18 years. The dmft ratio was 6.92 for 6–7-year-olds, and 4.72 for 8–9-year-olds, and the DMFT ratio was 6.57 for 12–14-year-olds, and 5.95 for those in the 15–18-year age group, respectively. No significant relationship between the patient groups during the temporary and permanent dentition periods was reported. The authors documented a high plaque score in 61.1% of the TM patients and described the patients as having poor oral hygiene status.

Recently, Al-Raeesi *et al*. (6) comparatively assessed the DMFT/dmft indices, oral hygiene index, occlusal anomalies, dentofacial and soft-tissue abnormalities in a total of 38 β-TM and 76 healthy Emirati children. The children with β-TM had a significantly higher DMFT and calculus index compared to the healthy controls, but a significantly lower gingivitis proportion.

In our study, the *S. mutans* counts, and the DMFT, plaque and oral hygiene indices of 59 β-TM and 50 control patients, aged between 6 and 16 years, were investigated. In accordance with Luglie *et al*. (9), the *S. mutans* levels in our study were significantly higher in the β-TM group when compared with the control group. The DMFT indices were significantly higher in β-TM patients than the control group, which concurred with the studies of Al-Raeesi *et al*. (6) and Al-Wahadmi *et al*. (10), but the data presented by Luglie *et al*. (9) were comparatively lower. Al-Wahadmi *et al*. (10), stated that DMFT ratios increased together with age in their study. In the current work, the plaque and oral hygiene index values were statistically higher in the β-TM patients, contrary to the research by Luglie *et al*. (9) and Al-Wahadmi *et al*. (10). The study carried out by Hattab *et al*. (8), reported that more than half of TM patients had a high plaque score. The differences between these studies and our work could originate from several parameters, such as the preferred study methods, and regional and socioeconomic factors.

*Lactobacillus*, which is responsible for dentin decay, was also examined in our study and no significant association occurred between the β-TM and control groups. According to the scanning results of available resources, no other studies have previously explored the saliva *Lactobacillus* densities of β-TM patients. There is a need for extensive studies to examine salivary *S. mutans* and *Lactobacillus* densities of β-TM patients.

When investigating the relationship between decay and gender in 47 TM patients, Leonardi *et al*. (18), noticed a higher decay prevalence in male TM patients. However, in Hattab *et al*. (11), and our study, a significant effect of gender was not determined.

Literature studies suggest the gingivitis occurrence frequency can be higher in TM patients than healthy individuals, and this can result from local factors or oral-maxillo-facial features of the disorder (9,11,12,19). In 2007, Ay *et al*. (20), compared to the blood lipid levels and periodontal parameters (gingival index, plaque index, probing bleeding, probing depth) in 24 TM patients with 20 age-matched control patients, and found that all periodontal parameters were significantly higher in the TM patients. Siamopoulou-Mavridou *et al*. (12), examined the gingivitis and decay experiences, as well as the flow rate, calcium, phosphorous, potassium, sodium, urea and lysozyme immunoglobulin levels (IgA, IgG, IgM) of the saliva of 21 TM and 83 control patients. According to the results, gingivitis and decay prevalence were statistically higher in TM patients. No significant difference was found in the flow rate of saliva while phosphorous and IgA were significantly lower in TM patients.

One of the aims of our study was to assess the decay tendency levels, by comparing saliva buffering capacities of β-TM and control patients, and no significant relationship was determined. To the best of our knowledge, no other current studies have analyzed the saliva buffering capacities of TM patients.

Multiple immune disorders can be seen in TM patients, which can create a predisposing effect for oral *Candida* (21-23). *Candida* types are opportunistic pathogens that exist in normal oral flora but can cause serious infections when the immune system is weakened. **Candida* albicans* is mentioned in the literature as the most isolated type, both in healthy people and TM patients (21-23).

In 2010, Hazza’a *et al*. (23) surveyed oral *Candida* carriers within 50 β-TM and 50 control patients by the mouthwash method. *Candida* was isolated in 74% (n=37) of the TM patients and 56% (n=28) of the control group, and the difference was significant.

In our study, *Candida* was isolated in 25.4% (15 patients) of β-TM patients and 34% (17 patients) of control patients and, according to our results, no significant relationship was evident between the study and control groups. Less *Candida* was isolated in β-TM patients in our study when compared with the study of Hazza’a *et al*. (23), probably due to differences in the methods used since Hazza'a *et al*. (23) preferred the mouthwash technique as the sample collection method, whereas, in our study, swab samples from the cheek and soft palate regions were collected. Socioeconomic, regional and other differences could be other factors contributing to the discrepancy.

To date, there are limited numbers of studies on oral *Candida* colonization of TM patients in the literature. Extensive studies are required, considering the occurrence possibility of *Candida*, among TM patients accompanied by multiple immune disorders.

The prevalence of β-TM patients in Antalya Province (West Mediterranean Region of Turkey) (12–13.1%) is above Turkeys’ average, so regular and strict treatment/follow-up protocols are carried out for β-TM patients in this region (24). This scenario allows envisaging the complications (e.g., oro-facial deformities, infections of the immune system, *Candida* infection) that can be experienced in β-TM patients. Educative and informative programs about treatment and follow-up of the disorder have been conducted regularly, for primary protection, as part of the treatment protocol.

As a conclusion, oro-dento-facial problems can be prevented before they occur, providing close examination by a dentist at diagnosis, treatment and follow-up of TM patients, beginning from childhood. Especially, pediatric dentists have a major preventive role in these problems. It is important that hematologists and pediatric dentists work together during treatment and follow-up procedures of TM patients. Informative and educative programs for TM patients and their parents must be provided by pediatric dentists, addressing complications which can occur in oro-dento-facial structures, as well as solutions and considerations.
